# Pygopus-2 promotes invasion and metastasis of hepatic carcinoma cell by decreasing E-cadherin expression

**DOI:** 10.18632/oncotarget.3570

**Published:** 2015-03-14

**Authors:** Sheng Zhang, Jie Li, Pingguo Liu, Jianfeng Xu, Wenxiu Zhao, Chengrong Xie, Zhenyu Yin, Xiaomin Wang

**Affiliations:** ^1^ Department of Hepatobiliary Surgery, Fujian Provincial Key Laboratory of Chronic Liver Disease and Hepatocellular Carcinoma (Xiamen University Affiliated ZhongShan Hospital), Xiamen, Fujian China

**Keywords:** pygopus-2, E-cadherin, hepatic carcinoma, invasion, metastasis

## Abstract

Pygopus-2 over-expression has been reported in several malignancies, such as ovarian, breast, lung and liver cancers. Here we demonstrated that down-regulation of Pygopus-2 by shRNA inhibited hepatic carcinoma cell invasion in vitro and metastasis in xenograft tumor models, which were promoted when Pygopus-2 was over-expressed. Pygopus-2 increased hepatic carcinoma cell invasion and metastasis, by decreasing E-cadherin. Pygopus-2 could bind to the E-cadherin promoter, increasing its methylation, and also indirectly decreased zeb2 expression. In turn these effects caused down-regulation of E-cadherin, potentiating invasion and metastasis. We suggest that targeting Pygopus-2 may potentially inhibit metastasis of hepatic carcinoma.

## INTRODUCTION

Hepatic carcinoma (HCC) is the sixth common neoplasm in the world [1]. The clinical efficacy remains un-satisfactory, despite long-range researches on HCC therapy and the great advance achieved in diagnosis. Recurrence and metastasis are still the main reason for many patients died within three year after the clinical therapy [2] and the 5-year survival rate is no more than 30% [3]. Therefore, it is of great importance to search effective prediction and valuable markers to precisely protect HCC patients away from recurrence and metastasis.

Dysregulation of canonical Wnt/β-catenin signaling pathway is associated with the development and progression of many malignancies, including HCC [4, 5]. In the last few years, growing body of evidences indicated that Pygopus (Pygo) was an important co-activator of the Wnt/β-catenin transcriptional complex in Drosophila [6] and acted as an enhancer of β-catenin function [7, 8]. Subsequently, Pygopus-2 (Pygo2) as a homologue of Pygopus was also detected in mammalians [9]. The biological function of Pygo2 includes regulating mammary gland development and stem/progenitor cell expansion, lens development [10], hair density [11], and so on. In addition, Pygo2 over-expression has been reported in ovarian [12], breast [13], cervical [14] and lung [15] cancers. Increased expression of nuclear Pygo2 has been reported in 82% of epithelial ovarian cancer tissues [12], and silencing of endogenous Pygo2 can not only suppress the growth of breast and epithelial ovarian cell lines [13] but also induce apoptosis of lung cancer cell lines [16]. What's more, our previous study has found that Pygo2 mRNA and protein expression was significantly higher in HCC tissues, abnormal high expression of Pygo2 protein in HCC patients correlated with a poor prognosis [17]. Obviously, Pygo2 maybe play an important role in HCC development and progression. However, little is known about Pygo2 functions in HCC cell invasion and metastasis.

E-cadherin is a calcium-dependent cell-cell adhesion protein and loss of E-cadherin has been linked with tumor metastasis, invasion, and poor prognosis [18-21]. If E-cadherin is down-regulated, the epithelial cells acquire a fibroblastoid morphotype and accompanied with the acquisition of invasive and metastasizing ability [22, 23]. Furthermore, zeb2, which is well-known to repress E-cadherin transcription directly, was over-expression in many tumors [24, 25]. However, to the best of our knowledge, there are no studies regarding the relationship between Pygo2 and E-cadherin expression, and zeb2 as well in HCC.

In order to examine the potential role of Pygo2 expression on tumor cells invasion and metastasis, we established HCC cell lines by shRNA and plasmid construction methods in the present study. We here revealed that Pygo2 could positively regulate HCC cell metastasis and invasion ability via repression of E-cadherin mRNA and protein expression, not only by promoting its promoter methylation directly but also via up-regulating zeb2 indirectly. These findings associates Pygo2 with metastasis in HCC, and provide a potential therapeutic target for treating HCC as well.

## RESULTS

### Knockdown of Pygo2 inhibits HCC cell invasion and migration, whereas ectopic expression of Pygo2 promotes invasion and migration of HCC cells *in vitro*

**Figure 1:**
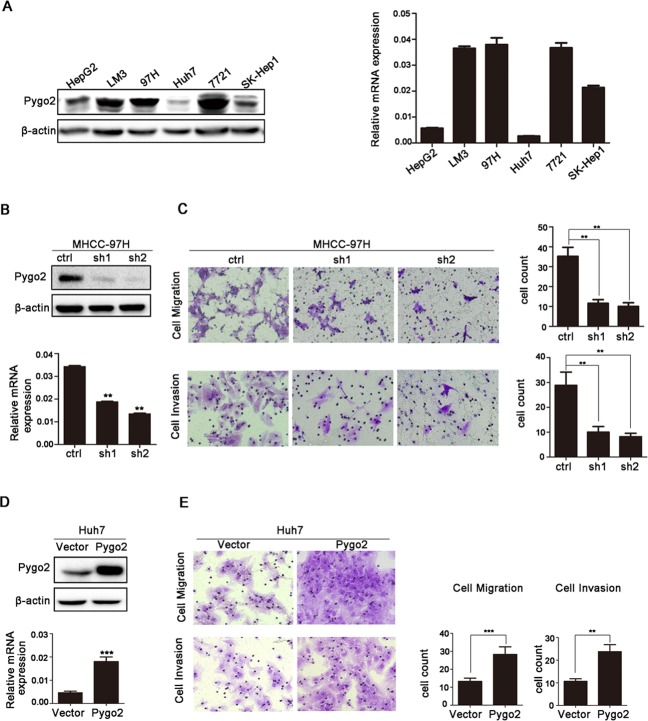
Pygo2 positively regulates invasion and migration of HCC tumor cells *in vitro* (A) Pygo2 protein and mRNA expression pattern in human HCC cell lines. (B) Pygo2 protein level was dramatic decreased in 97H-shPygo2 cells. (C) Down-regulation of Pygo2 repressed MHCC-97H cell invasion and migration. (D) Pygo2 protein was increased in Huh7-overexpression Pygo2 cells. (E) Over-expression of Pygo2 promoted Huh7 cell invasion and migration. **P ≤ 0.01, ***P ≤ 0.001.

Our previous study found that Pygo2 was abnormal expression in HCC tissues [17]. In the present study, we examined Pygo2 expression pattern in some HCC cell lines including HepG2, LM3, MHCC-97H, Huh7, SMMC-7721 and SK-Hep1. Expression of Pygo2 was detected in these cells with different protein and mRNA levels (Fig. 1A). Relative higher Pygo2 expression was detected in MHCC-97H, SMMC-7721 and lower in HepG2 and Huh7, respectively. Interestingly, our previous study has found that the metastatic potential of the MHCC-97H and SMMC-7721 cells was remarkably greater than that of the HepG2 cells [26]. In addition, Pygo2 over-expression in HCC correlates with patients' intra- and extra-hepatic metastasis (P = 0.029) and vascular invasion (P = 0.026) [17]. Collectively, the above results implied that Pygo2 protein abnormal expression might be associated with HCC cell invasion and metastasis.

In order to clarify this hypothesis, we stably neutralized endogenous Pygo2 using a lentivirus vector-based shRNA approach in MHCC-97H cells. The protein and mRNA level of Pygo2 was dramatically down-regulated in 97H-ShPygo2 compared with 97H-shCtrl (Fig. 1B). Migration and invasion assay showed that down-regulation of Pygo2 significantly attenuated cell migration and invasiveness capability (Fig. 1C). However, when Pygo2 plasmid was cloned into a lentiviral vector and stably transfect into Huh7 cell lines, invasion and migration assay showed the opposite results (Fig. 1D, 1E). Collectively, our data indicated that Pygo2 expression was positively associated with HCC cell invasion and metastasis *in vitro*.

### Pygo2 can regulate E-cadherin mRNA and protein expression in HCC cells

**Figure 2:**
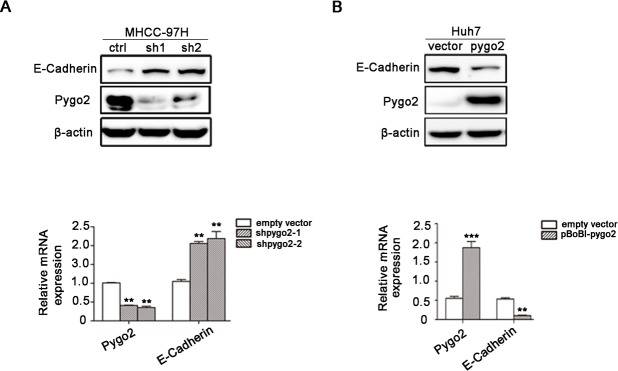
Pygo2 can regulate E-cadherin mRNA and protein expression in HCC cells (A) The protein and mRNA level of E-cadherin were up-regulated in 97H-shPygo2 cells compared with 97H-shCtrl. (B) E-cadherin mRNA and protein level were decreased significantly when Pygo2 was over-expression in Huh7 cells. **P ≤ 0.01, ***P ≤ 0.001.

In the present study, Pygo2 was shown to positively regulate HCC cell migration and invasion. However, the underlying molecular mechanism remains unclear. E-cadherin was selected in our further research because of its biological function on tumor metastasis [27] and association with HCC carcinogenesis [28]. To examine whether E-cadherin is regulated by Pygo2 in HCC cells, we tested the expression pattern of E-cadherin in Pygo2 down-regulated cell lines by Real-time PCR and Western Blot methods. Results showed that expression of E-cadherin mRNA and protein were significantly increased in 97H-ShPygo2 compared with 97H-ShCtrl (Fig. 2A). On the contrary, when Pygo2 plasmid was stably transfect into Huh7 cells, expression of E-cadherin mRNA and protein level were decreased significantly (Fig. 2B).

### Pygo2 and E-cadherin are inversely correlated in human liver cancer tissues

**Figure 3:**
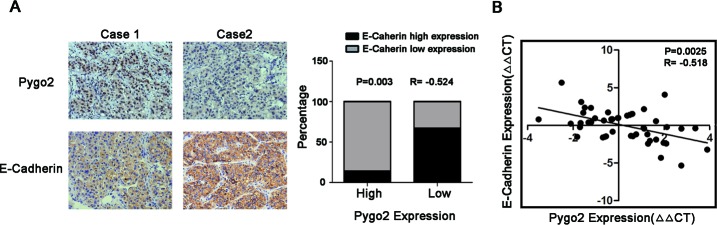
Pygo2 and E-cadherin are inversely correlated in human liver cancer tissues (A) Immunohistochemical staining of Pygo2 and E-cadherin in HCC tissues, and Spearman's correlation analysis showed that Pygo2 expression was negatively correlated with E-cadherin. (B) Pygo2 mRNA level in HCC tissues was negative associated with E-cadherin.

Base on our experiment finding on HCC cell lines that down-regulated of Pygo2 can increase E-cadherin expression, we wonder if their relationship hold true in hepatic cancer tissues. Immunohistochemistry experiment was performed on 30 cases of HCC tissues and results showed that the high level of Pygo2 expression was associated with a low level of E-cadherin. Inversely, lower expression of Pygo2 was accompanied with a higher expression of E-cadherin protein. Spearman's rank correlation analysis confirmed the negative correlation between Pygo2 and E-cadherin (P = 0.003, r = − 0.524, Fig. 3A). In addition, we used Real-time PCR to detect the mRNA expression pattern and found the similar results that Pygo2 mRNA level was negative associated with E-cadherin (P = 0.0025, r = −0.518, Fig. 3B). Together, these results suggested that E-cadherin was negatively regulated by Pygo2 not only exist in HCC cell lines, but also occur in clinical samples.

### Pygo2 induces HCC cells invasion via regulation of E-cadherin

**Figure 4:**
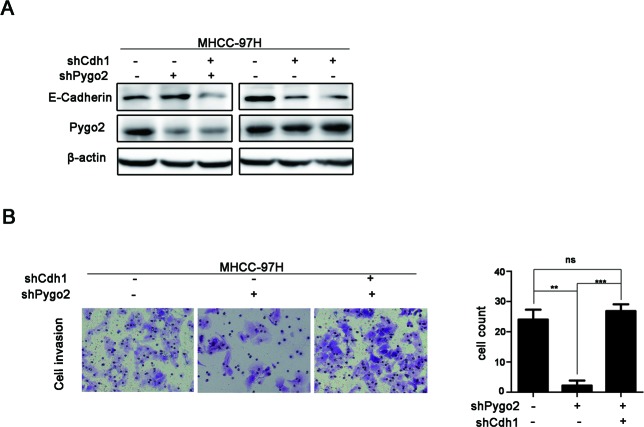
Pygo2 induces metastasis via regulation of E-cadherin (A and B) Pygo2 down-regulation induced weaken invasiveness of MHCC-97H cells was strongly restored by the repressed of E-cadherin. **P ≤ 0.01, ***P ≤ 0.001.

Since Pygo2 can promote HCC cell invasion, and regulate E-cadherin protein level in our present study. Whether E-cadherin down-regulation is required for Pygo2 induced HCC cells invasion. In order to investigate this suppose, trans-well study was performed in 97H-shCtrl, 97H-shPygo2 and 97H-shPygo2+shCdh1 cells. As seen in Fig. 4A and 4B, the down-regulation of Pygo2 in 97H-shPygo2 markedly weaken the invasiveness of MHCC-97H cells. However, Pygo2 down-regulation induced weak invasiveness was strongly restored by the repressed of E-cadherin expression. Taken together, these results indicated that E-cadherin is required for the regulation of invasion by Pygo2.

### Pygo2 can binds to E-cadherin promoter

**Figure 5:**
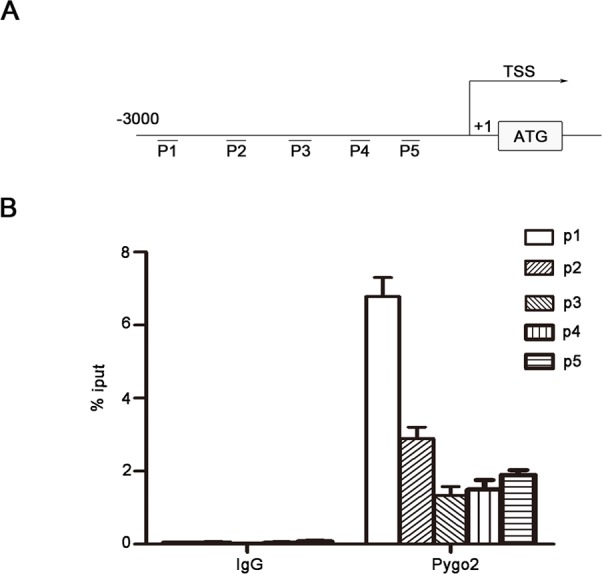
Pygo2 binds to E-cadherin promoter directly (A) We designed 5 paired random primers for E-cadherin promoter. (B) The primers set resulted in a strong DNA amplification of the E-cadherin promoter region compared with control, which were detected by Real-time PCR method.

Gu Bingnan et al. has found that Pygo2 is located in cell nuclear and contain a conservative PHD domain which can binds to multiple histone promoters [29]. Pygo2 was mainly located in HCC cell nuclear and could up-regulate E-Cadherin mRNA level, it is possible that Pygo2 can bind to the promoter of E-cadherin and regulate its gene expression. To test this hypothesis, binding of endogenic Pygo2 protein to the E-cadherin promoter was analyzed *in vitro*. MHCC-97H cells were cultured in conditioned medium and then subjected to chromatin immunoprecipitation experiment by using a rabbit monoclonal antibody against Pygo2 (sc-98744, Santa Cruz) and a rabbit IgG antibody as a control. As showed in Fig. 5, we found that the primers set especially primer 1, resulted in a strong DNA amplification of the E-cadherin promoter region. Collectively, chromatin immunoprecipitation results indicated that Pygo2 could directly bind to E-cadherin promoter.

### Pygo2 regulates E-cadherin expression not noly via binding to and promoting its promoter methylation directly, but also by up-regulating zeb2 indirectly

**Figure 6:**
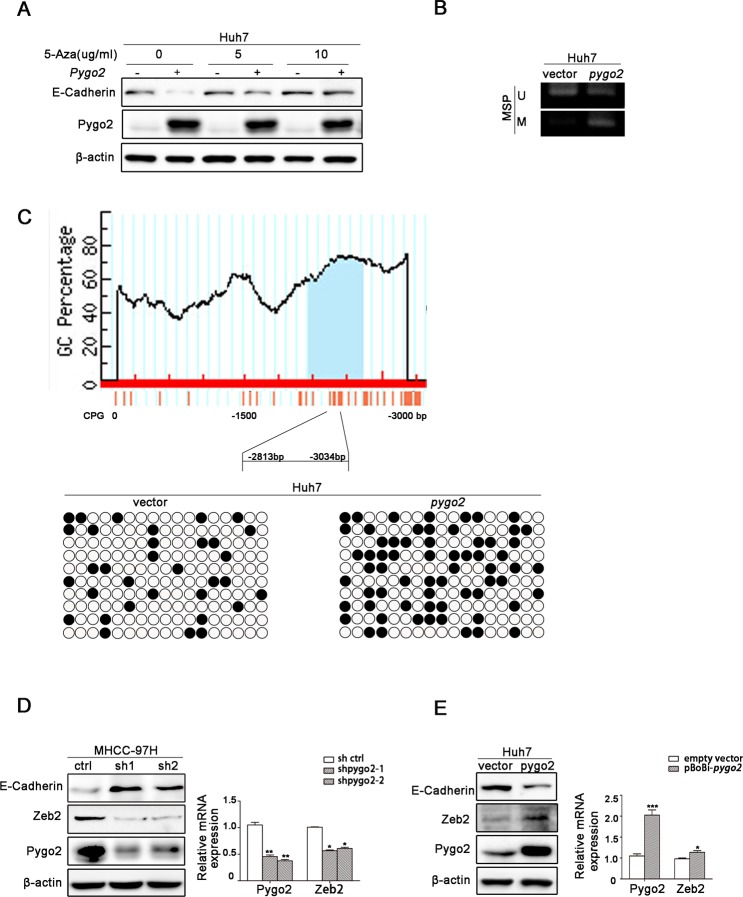
Pygo2 regulates E-cadherin expression not noly via binding to and promoting its promoter methylation directly, but also by up-regulating zeb2 indirectly (A) E-cadherin protein level was increased accompany by the addition with 5-azacytidine in Huh7 cells, compared with lanes 1, 3 and 5. Treatment of Huh7-overexpression Pygo2 cells with 5Aza partially restored expression of E-cadherin, compared with lanes 2, 4 and 6. (B) MSP assay showed that Pygo2 could promote E-cadherin promoter CpG island methylation. (C) Bisulfite sequencing PCR assay confirmed that Pygo2 could promote E-cadherin promoter methylation. (D) Zeb2 protein and mRNA level were decreased in 97H-shPygo2 cells compared with Ctrl cells. (E) Zeb2 protein and mRNA level were increased when Pygo2 was over-expressed in Huh7 cells. *P ≤ 0.05, **P ≤ 0.01, ***P ≤ 0.001.

Many studies have demonstrated that promoter methylation of E-cadherin is an important mechanism contributing to its down-regulation [30]. We used a methyltransferase inhibitor 5-azacytidine (5Aza) to treat Huh7 cell lines and found that E-cadherin level was increased accompany with the addition of 5-azacytidine (Fig. 6A). This suggests that E-cadherin transcription in our present cell models was manipulated by methylation regulation. Then we speculate methylation of the E-cadherin promoter maybe play an important role in Pygo2-mediated decreasing of this gene. Thus we treated Huh7-overexpression Pygo2 cells with or without 5Aza. As shown in Fig. 6A, treatment of Huh7-overexpression Pygo2 cells with 5Aza partially restored the expression of E-cadherin. Next, methylation analysis of the E-cadherin promoter showed that the CpG island methylation status of Cdh1 (E-cadherin) in Huh7-overexpression Pygo2 was dramatically more than control cells (Fig. 6B). And these results were confirmed by Bisulfite sequencing PCR assay (Fig. 6C). Together, these results suggest that Pygo2 can promote the methylation of E-cadherin promoter.

Zeb2, as a transcription factors, has been detected in many cancers, and it is well-known to repress E-cadherin transcription via directly binding to the E-boxes within the E-cadherin promoter [31, 32]. And Chu-an Wang et al. found that zeb2 can enhance E-cadherin promoter methylation level [25]. Since Pygo2 can negatively regulate E-cadherin mRNA expression, whether there is a relationship between Pygo2 and zeb2. In order to clarify this speculation, we used Western blotting to examine the levels of zeb2 in HCC cells and found that zeb2 protein was significantly reduced when Pygo2 was down-regulated. The mRNA levels of zeb2 was also measured by Real-time PCR and showed a similar result (Fig. 6D). Inversely, zeb2 protein and mRNA level were increased in Huh7 over-expression Pygo2 cells compared with Ctrl (Fig. 6E). Collectively, these results above demonstrated that Pygo2 repress E-cadherin mRNA and protein expression, not only by binding to and promoting E-cadherin promoter methylation directly but also via up-regulating zeb2 indirectly.

### Knockdown of Pygo2 inhibits HCC metastasis *in vivo*

**Figure 7:**
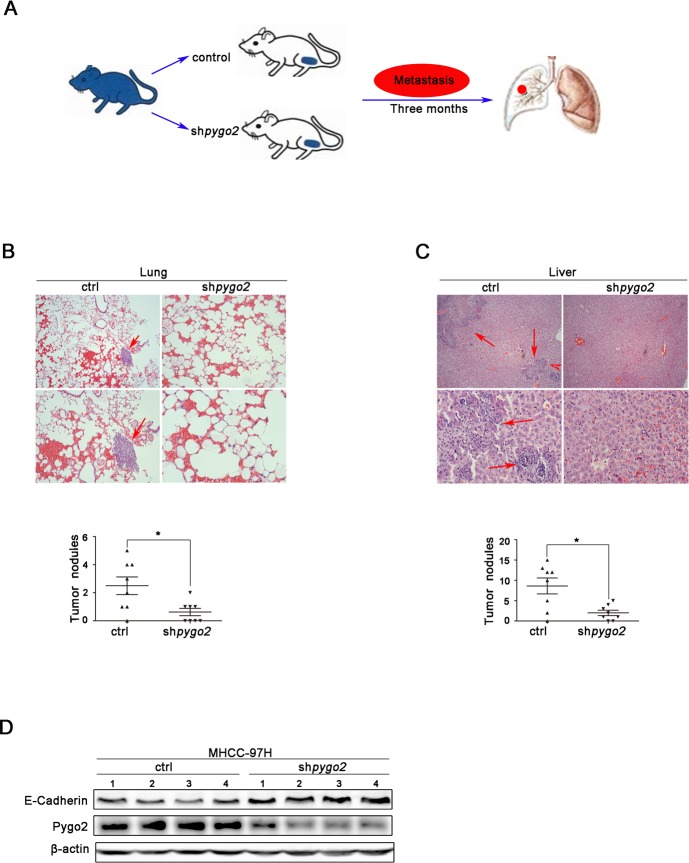
Pygo2 enhances HCC metastasis *in vivo* (A) Nude mouse xenograft model was constructed via the subcutaneous of nude mouse injection with 97H-shPygo2 and 97H-shCtrl cells. (B) The metastatic nodules in 97H-shCtrll group lungs were less than 97H-shCtrl models. (C) The metastatic nodules in 97H-shCtrll group livers showed the same results. (D) Western blot analysis of the expression of Pygo2 and E-cadherin protein in 97H-shPygo2 and 97H-shCtrl tumors. *P ≤ 0.05.

We further want to know whether down-regulation of Pygo2 has a uniform effect *in vivo*, a xenograft model was constructed via the subcutaneous of nude mouse injection with 97H-shPygo2 and 97H-shCtrl cells (Fig. 7A). Three months after implantation, tested mice were sacrificed and the metastatic nodules were counted. The mice injected with 97H-shPygo2 cells displayed less pulmonary and intra-hepatic micro-metastases nodules which were confirmed via histological study and hematoxylin and eosin stained compared with the mice injected with 97H-shCtrl (Fig. 7B & 7C). In order to correlate the biological response with the mechanisms identified in the HCC cells system, E-cadherin protein levels were assessed by western blot analysis. As shown in Fig. 7D, knockdown of Pygo2 significant increased E-cadherin protein level in transplanted tumor tissue. All above suggest that knockdown of Pygo2 could inhibit HCC metastasis *in vivo*.

## DISCUSSION

Dysregulation of Wnt signaling pathway and epigenetic modification are both associated with HCC tumorigenesis. Pygo2 as an important component of Wnt signaling pathway, it functions in targeting Armadillo/β-catenin to the nucleus [33]. In addition, Pygo2 can facilitates histone methyltransferase (HMT) and histone acetyltransferase (HAT) interaction with β-catenin and further augment Wnt1-induced, TCF/LEF-dependent transcriptional activation in breast cancer cells [34]. Molyneux et al. found that stem cells in the mammary epithelium are involved in tumor development [35] and Pygo2 can also regulate proliferation of both embryonic mammary progenitors and postnatal mammary stem cells of the terminal end bud [36]. Hence, it is easy to comprehend that Pygo2 acts as a connection among epigenetics, Wnt signaling, and stem cells [37].

What's more, abnormal Pygo2 expression has been reported in breast cancer [13], lung cancer [15, 16], and glioma [38]. In addition, abnormal expression of Pygo2 in cancers not only associated with tumor growth and apoptosis [39], but also has an important clinic-pathological significance. However, litter is known about the biological function of Pygo2 in HCC. Despite our previous research has found that Pygo2 was over-expressed in human liver cancerous tissues and associated with a poor prognosis of HCC patients, whether there is a relationship between Pygo2 abnormal expression and HCC cell invasion and metastasis which plays important roles in resulting in a poor prognosis rather than tumorigenesis is still unclear. In the present study, we applied shRNA-mediated Pygo2 depletion technology to down-regulate endogenic Pygo2 in MHCC-97H cells and demonstrated for the first time that weaken Pygo2 expression can inhibited the invasion of live cancer cells and metastasis *in vitro*. When Pygo2 plasmid was cloned into a lentiviral vector and stably transfected into Huh7 cell lines, invasion and *in vivo* assay showed the opposite results. Therefore, these results can explain our previous findings that Pygo2 over-expression in HCC tissues correlated with extra-hepatic metastasis and vascular invasion [17].

E-cadherin is a core protein mediated cell-cell adhesion to hold the epithelial cells tight together. Loss of E-cadherin decreases the cellular adhesion, resulting in an increase of cell motility [40]. In order to investigate the mechanism of Pygo2 regulation on HCC cell invasion, functional study found that E-cadherin mRNA and protein levels were down-regulated in Pygo2 over-expressed cells and up-regulated when endogenous Pygo2 was silenced by shRNA technology. In the clinical HCC tumor samples, Pygo2 is also negatively correlated with the expression of E-cadherin. However, Pygo2 protein level was not change when E-cadherin was down-regulated in Huh7 cells by shRNA method (Fig. 4A), which demonstrated that E-cadherin was a downstream target of Pygo2. What's more, Sh-Cdh1 in 97H-shPygo2 cells experiment revealed that Pygo2 induces HCC cells invasion via repressing of E-cadherin expression.

**Figure 8:**
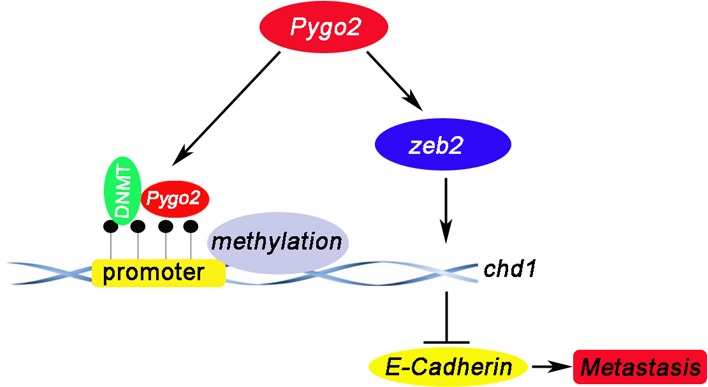
A proposed model depicting the mechanism by which Pygo2 represses E-cadherin expression and promotes metastasis of HCC cells Pygp2 repress E-cadherin expression not only via binding to E-cadherin promoter and promoting its promoter methylation directly, but also by up-regulating Zeb2 indirectly, and subsequently promoting HCC metastasis progress.

Our previous research has found that 66.7% of the HCC tumors showed nuclear accumulation of the Pygo2 protein, nuclear expression of Pygo2 was more frequent in HCC tissues than normal. Maybe there was a combination between Pygo2 and E-cadherin gene promoter. Interestingly, chromatin immuno-precipitation assays confirmed our speculation and found that Pygo2 could really bind to E-cadherin promoter directly. What's more, methylation assay results showed that Pygo2 can not only bind to E-cadherin promoter, but also promote its promoter methylation. This maybe the main reason of Pygo2 negatively regulate E-cadherin expression. In addition, up-regulation of many factors (zeb2, kras, slug and snail) has also been reported in many cancers, which were well known to inhibit E-cadherin expression via different pathway. In order to found out other mechanism by which Pygo2 negatively regulate E-cadherin expression in our present study, we examined whether Pygo2 can alter any of these above factors by Real-Time PCR methods. Of these known factors, only zeb2 mRNA levels dramatically changed both in 97H-shPygo2-1 and 97H-shPygo2-2 cells compared with control group (Supplementary Fig. S1). Whereas Sánchez-Tilló E et al. have found that zeb2 repress Cdh1 (E-cadherin) via binding directly to E-boxes within E-cadherin promoter [32]. Collectively, we draft a possible work model in which Pygo2 enhances HCC cell invasion and migration at least partially by repressing E-cadherin expression. Pygo2 can bind to E-cadherin promoter directly and inhibit E-cadherin transcription by promoting its promoter methylation. In addition, Pygo2 suppress E-cadherin transcription by up-regulating zeb2 indirectly; thus inhibits E-cadherin both via transcription and epigenetic mechanism (Fig. 8).

Since epithelial-mesenchymal transition (EMT) is one of the crucial events regulating HCC tumor invasion and metastasis [41, 42], cancer cells that undergo EMT process can get a abilities of invasion and metastasis [43]. The effect of Pygo2 on EMT was also detected in the present study. Three mesenchymal markers (N-cadherin, Fibronectin and MMP-2) were changed in Pygo2 over-expressed and silenced cells, suggesting that the invasive and metastatic effect of Pygo2 on HCC cell was via inducing EMT as well (Supplementary Fig. S2). However, we may be found out other possibility that Pygo2 influence HCC cell invasion and metastasis by our further study.

In summary, Pygo2 plays an important role in HCC invasion and metastasis through repressing E-cadherin expression via transcription and epigenetic mechanism, and subsequently inducing EMT progress. Consequently, targeting Pygo2 could be a useful therapeutic strategy for the prevention of HCC patients away from recurrence and metastasis.

## MATERIALS AND METHODS

### Cell culture

Human liver cancer cell lines HepG2, LM3, MHCC-97H, Huh7, SMMC-7721 and SK-Hep1 were purchased from the cell bank of Shanghai Institute of Cell Biology (Shanghai, China) and cultured in either DMEM or RPMI 1640 mediums (HyClone, Logan, UT, USA) supplemented with 10% fetal calf serum (Biological Industries, Shanghai, China), and with 100 IU/ml penicillin and 100 μg/ml streptomycin (Sigma, St. Louis, MO, USA). Cells were grown on sterile culture dishes and passaged every 2-4 days using 0.25% trypsin (Invitrogen, Carlsbad, CA, USA).

### Patients and specimens

All of the clinical samples were obtained from Chronic Liver Disease Biological Sample Bank, Department of Hepatobiliary Surgery, Zhongshan Hospital Xiamen University. Specimen collection was performed after obtaining informed consents from each patient, and the study was approved by the Ethics Committee of the hospital.

### Plasmid construction and lentivirus preparation

For target gene knockdown, control and the two shRNA sequences against Pygo2 (sequences were shown in Table 1) were sub-cloned into the pSIREN-RetroQ-puro RNA interference vector downstream from the U6 promoter. For Pygo2 over-expression, 1221 bp genomic sequence of Pygo2 coding region was cloned into the backbone of PBOBI-CMV vector downstream from the CMV promoter. Then 293 (T) cells were transfected with the above mentioned plasmids and the virus packaging plasmids Vsvg and PHR using the Tubofect transfection reagent (thermo Cat #R0531, R0532). The virus-containing supernatant was harvested 48 hours post-transfection, filtered to remove cell debris, and used for infection after titer quantification. Cell lines were plated in six wells plates with full conditional media for 24h prior to infection. Infection was performed via virus-containing supernatant and Polybrene (10ug/ml). Infected cells were cultured for selection with puromycin (1.2ug/ml) after infecting twice. Stable transfectants were maintained in regular medium with puromycin (1.0ug/ml) for further analysis.

**Table 1:** Primers sequences

RT-PCR	Primer name	F:5′-3′	R:5′-3′
	Pygo2	CCTTCTCTGTCCCAACGATTT	CCAGGAAAGGGACTTGTGTTAG
	E-Cadherin	AGCCATGTACGTTGCTATCC	CGTAGCACAGCTTCTCCTTAAT
	β-actin	ATAGCACAGCCTGGATAGCAACGTAC	CACCTTCTACAATGAGCTGCGTGTG
	Zeb2	CCCATTCTGGTTCCTACAGTTC	GGGAAGAACCCGTCTTGATATT
	Slug	AACTACAGCGAACTGGACAC	GAGGATCTCTGGTTGTGGTATG
	Snail	CAGATGAGGACAGTGGGAAAG	GAGACTGAAGTAGAGGAGAAGGA
	Kras	CAAGAGTGCCTTGACGATACA	GACCTGCTGTGTCGAGAATATC
shRNA	Pygo2-1	GATCCGCCTGCATACTCACATCTGAT TCAAGA-GATCAGATGTGAGTATGCAG GTTTTTTACGCGTG	AATTCACGCGTAAAAAACCTGCATAC TCACATCTGATCTCTTGAATCAGATGT GAGTATGCAGGCG
	Pygo2-2	GATCCGCTCACATCTGACGGAGTTTTTC AAGAGAAAACTCCGTCAGATGTGAGTT TTTTACGCGTG	AATTCACGCGTAAAAAACTCACATCTG ACGGAGTTTTCTCTTGAAAAACTCCGT CAGATGTGAGCG
	E-Cadherin	GATCCGCACCAAAGTCACGCTGAATTTC AAGAGAATTCAGCGTGACTTTGGTGTTT TTTACGCGTG	AATTCACGCGTAAAAAACACCAAAGTC ACGCTGAATTCTCTTGAAATTCAGCGTGA CTTTGGTGCG
Pbobi-cmv	Pygo2	GCTCTAGAATGGATTACAAGGATGACGA CGATAAGGCCGCCTCGGCGCCGCCCCCA	CCCTCGAGTCACCCATCGTTAGCAGCC AC
Chip-RT-PCR	E-Cadherin-P1	CAGTTGCTATGATGAGCCAAGA	GGGAAGTCAGTGTTCTCCTTTG
	E-Cadherin-P2	CTCTCATTGGCCTCAATCTCTC	GCCACTGACCAGCTCATTTA
	E-Cadherin-P3	ACCACGCCTGGCTAATTT	GATCACGAGGTCAGGAGATTG
	E-Cadherin-P4	CTCACTAACCCATGAAGCTCTAC	GCCGAGGCTGATCTCAAAT
	E-Cadherin-P5	CACCTGTACTCCCAGCTACTA	GGTCTCACTCTTTCACCCAAG
Methylation	MSP for E-Cadherin Promoter-M	TGGGTAAGATAGAGCGAGATTTC	TCTCGAACTCCTAAACTAAAACGAT
	MSP for E-Cadherin Promoter-U	GGGTAAGATAGAGTGAGATTTTGT	TCTCAAACTCCTAAACTAAAACAAT
	BSP for E-Cadherin Promoter	TTTTAGTAATTTTAGGTTAGAGGGTTAT	AAACTCACAAATACTTTACAATTCC

### Hematoxylin-eosin and immunohistochemistry staining

Tissues were fixed with 10% neutral formalin, embedded in paraffin, and 3-μm-thick sections were prepared by pathological technologist. Hematoxylin-eosin (HE) stain was performed as previous describe [17]. For immunohistochemistry (IHC) staining, sections were deparaffinized, hydrated and soaked in 3% H_2_O_2_ for 15 minutes at room temperature, and then incubated with Pygo2 polyclonal antibody (1:4000, ab109001, Abcam) and E-cadherin (1:1000, 20874-1-AP, Proteintech) at 4°C overnight. Biotinylated secondary antibody and diaminobenzidine were purchased from Maixin Biotechnology (Fuzhou, China). Evaluation of Pygo2 and E-cadherin staining in HCC tissue sections was performed based on the IHC assessment methods used by Popadiuk et al. for epithelial ovarian cancer [12] and Motoyuki Hashiguchi et al. for HCC [44], respectively.

### Western blot analysis

Total protein was extracted with RIPA lysis buffer supplemented with Protease Inhibitor Cocktail (Sigma, St Louis, MO, USA) and quantified using the Bradford method. Protein lysate was loaded on 10-12% sodium dodecyl sulfate polyacrylamide gel electrophoresis and transferred to the polyvinylidene fluoride membrane. After blocking the membranes with non-fat milk, the blots were incubated with primary antibodies directly against Pygo2 (1:1000, ab109001, Abcam), β-Actin (1:1000, Cell signaling technology), E-cadherin (1:800, 20874-1-AP, Proteintech), Zeb2 (1:500, 14026-1-AP, Proteintech), Fibronectin (1:1000, Cell signaling technology), N-cadherin (1:1000, Cell signaling technology), MMP-2 (1:1000, Cell signaling technology) at 4°C overnight. After that, the blots were incubated with the secondary antibody labeled with horse radish peroxidase at room temperature for 2 h. Protein bands were visualized using enhanced chemiluminescence and quantitated by densitometry using Image-J software. The relative protein levels were calculated by comparison to the amount of β-Actin protein. Experiments were repeated in triplicate.

### Real-time PCR

RNA was extracted from tissues or cells samples using the Trizol reagent (Ambion Cat. 15596-026, USA) according to the manufacturer's instructions. Primers were designed and synthesized by BGI-Tech. The sequences of the primer pairs were showed in Table 1. A dissociation procedure was performed to generate a melting curve for confirmation of amplification specificity. β-actin mRNA was quantified in parallel as the reference control. Relative gene expression was calculated using the 7500 System SDS software. All experiments were repeated three times independently.

### Matrigel invasion assay

The invasion assay was performed as reported previously with some modifications [45]. The cells' invasive and migration abilities were examined using a 24-well trans-well with 8-μm pore polycarbonate membrane inserts (Becton Dickinson, Franklin Lakes, NJ, USA) according to the manufacturer's protocol. 2× 10^6^ cells were planted on the upper chamber. After 48h in culture, cells that appeared on the lower surface of the filter were fixed with 100% methanol and stained with crystal violet and counted in five random ×200 fields using an inverted microscope and with the double-blind method. The experiments were performed in triplicate and got means were calculated.

### Chromatin immunoprecipitation

The chromatin immunoprecipitation (ChIP) assay was performed using an EZ-ChIP kit (Millipore, Catalog No. 17-10461) according to manufacturer's instructions. The E-cadherin promoter region located −3000 to −1 bp upstream of the transcription start site was amplified, and products were quantified by real-time PCR using both the ChIP-enriched DNA and input DNA as template. Enrichment by ChIP was assessed relative to the input DNA and normalized to the level of β-actin. The PCR primers for E-cadherin were listed in Table 1.

### Methylation analysis

Genomic DNA (0.5 μg) was treated with CpGenome™ Direct Prep Bisulfite Modification Kit (millipore, Catalog No. 17-10451) according to the manufacturer's protocol. Bisulfite-converted DNAs (~50 ng) were used as templates for PCR amplification of the CpG islands in the Cdh1 promoter. All PCR products were purified from 2% agarose gels using a Gel Extraction Kit (TAKARA. CAT#9762) and cloned into the pMD18-T vector (TAKARA, CAT#6011). Ten randomly selected clones from each sample were selected for sequencing. MSP was performed on bisulfate-modified DNA according to manufacturer's protocol.

### Animal studies

For the *in vivo* metastasis assays, 2 × 10^5^ cells were injected subcutaneous of armpit into 4 to 5 weeks old nude mouse (8 cases for 97H-shCtrl group and 97H-ShPygo2 group respectively). The mice were sacrificed 12 weeks later and all visceral organs or tissues that are possibly with metastasis such as lung, liver and intestinal tract were collected for metastatic foci examination and standard pathological study. In short, the tissues were fixed with 10% neutral formalin and tissues blocks were cut into 3 μm sections and further for HE stain. Animal work was performed on an approved Institutional Animal Care and Use Protocol of Xiamen University.

### Statistical analysis

All data were expressed as the mean ± SD and analyzed using the software of SPSS version 21.0. Statistical analysis was performed using two-related samples Wilcoxon nonparametric test for comparing two different groups. And the Spearman's rank correlation was used to examine possible correlations between Pygo2 and E-cadherin expression. Differences were considered statistically significant when P value was less than 0.05.

## SUPPLEMENTARY MATERIAL AND FIGURES


