# Supplementary material for: Pygopus-2 promotes invasion and metastasis of hepatic carcinoma cell by decreasing E-cadherin expression
Source: Oncotarget. 2015 Mar 14;6(13):11074–86. doi: 10.18632/oncotarget.3570 (PMC4484440; doi:10.18632/oncotarget.3570)
Supplement: Supplementary file 1 [file oncotarget-06-11074-s001.pdf]

Pygopus-2 promotes invasion and metastasis of hepatic carcinoma cell by decreasing E-cadherin expression

Supplementary Material

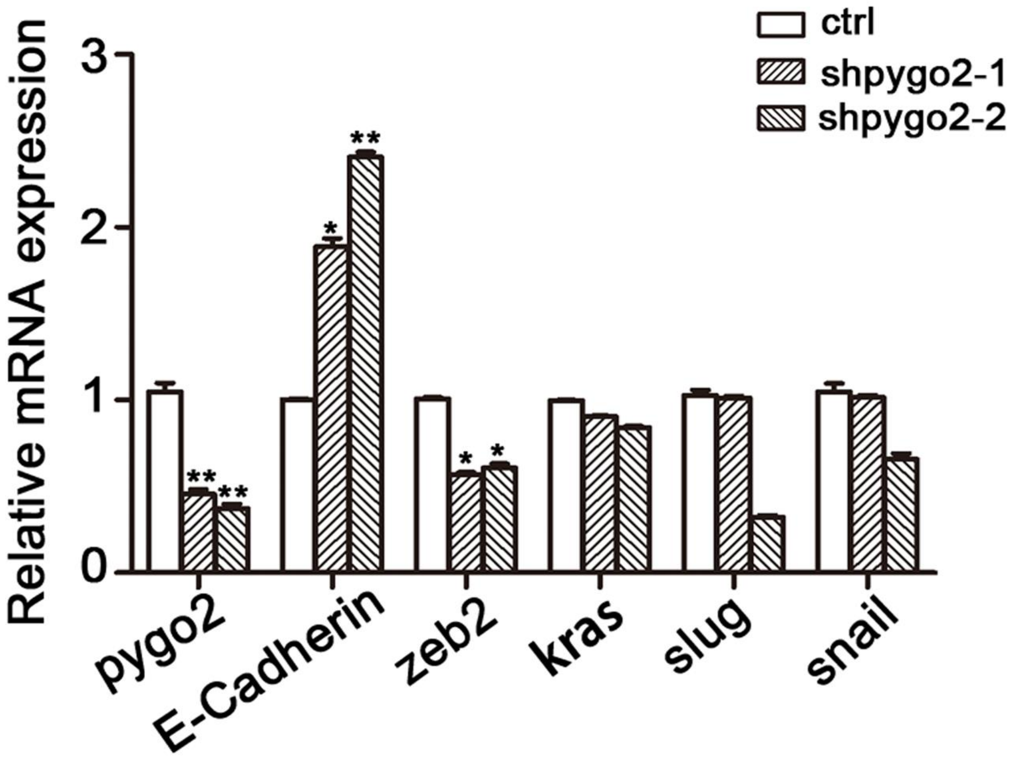

Figure S1: The effect of Pygo2 on the inhibitor of E-cadherin. Among the known inhibitor (zeb2, kras, slug and snail), only zeb2 mRNA levels showed altered expression both in 97H-shPygo2-1 and 97H-shPygo2-2 cells compared with control group. *$P \leqslant 0.05$, **$P \leqslant 0.01$.

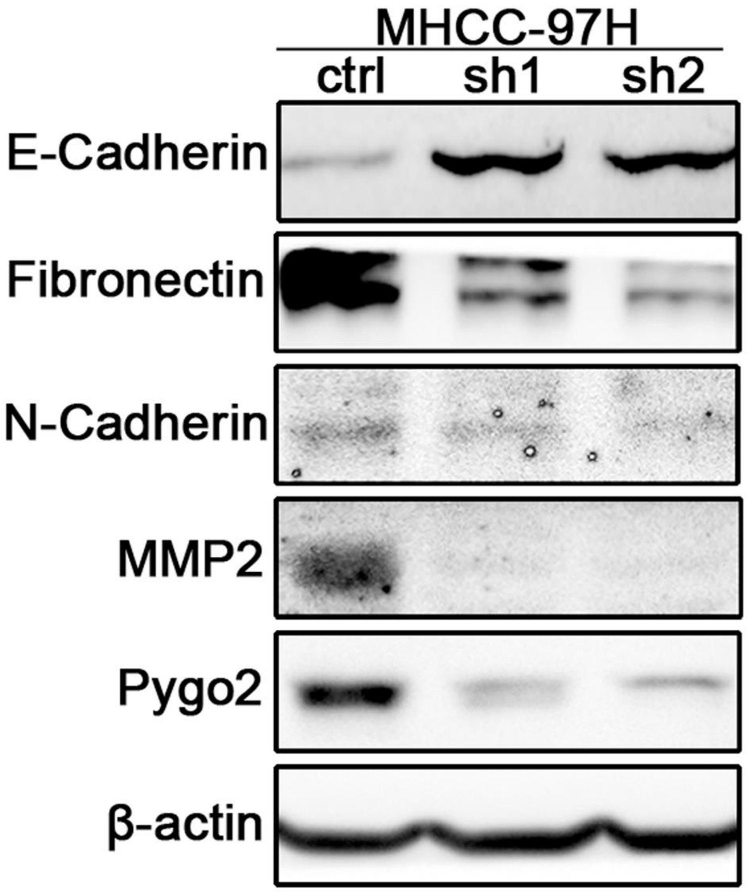

Figure S2: The effect of Pygo2 on EMT markers. The proteins level of N-cadherin, Fibronectin and MMP-2 were decreased when Pygo2 was down-regulated in 97H-ShPygo2.
